# The mediating role of anxiety and depression in the relationship between coping styles and life satisfaction among frontline medical workers during the COVID‐19 pandemic: A cross‐sectional study

**DOI:** 10.1002/ibra.12133

**Published:** 2023-09-25

**Authors:** Gui‐Fang Chen, Ye‐Ping Zhang, Zhi‐Jie Wei, Xin‐Lan Zhang, Jun Liu, Juan Peng, Zu‐Cai Xu, Chang‐Yin Yu, Jun Zhang

**Affiliations:** ^1^ Department of Psychiatry Zunyi Medical University Zunyi Guizhou China; ^2^ Department of Neurology Zunyi Medical University Zunyi Guizhou China

**Keywords:** COVID‐19, anxiety, coping style, depression, life satisfaction, mediation effect

## Abstract

This study aimed to examine the mediating role of anxiety and depression in the relationship between coping styles and life satisfaction among frontline medical workers during the COVID‐19 pandemic. Five hundred and fourteen frontline medical workers from Zunyi were recruited to complete questionnaires, including the Self‐rating Anxiety Scale (SAS), Self‐rating Depression Scale (SDS), Satisfaction with Life Scale (SWLS), and Simplified Coping Style Questionnaire (SCSQ). SPSS 24.0 was used to measure the characteristics of anxiety, depression, life satisfaction, and coping styles. We found that the prevalence rates of anxiety and depression among study participants were 22.57% and 18.29%, respectively. Besides, anxiety was positively correlated with depression; anxiety and depression were positively correlated with passive coping style but negatively correlated with life satisfaction and active coping style; life satisfaction was positively correlated with active coping style and negatively correlated with passive coping style (all *p* < 0.001). Moreover, anxiety and depression mediated the relationship between coping styles and life satisfaction. Anxiety accounted for 18.6% of the effect of active coping style and 35.48% of the effect of passive coping style on life satisfaction. Depression accounted for 48.84% of the effect of active coping style and 67.74% of the effect of passive coping style on life satisfaction. The present study provides novel insights into the effect of subclinical anxiety and depression on frontline medical workers in the pandemic area. Anxiety and depression yielded a mediating effect on the relationship between coping styles and life satisfaction.

## Graphical abstract

This study aimed to explore the mediating role of anxiety and depression in the relationship between coping styles and life satisfaction in medical workers during the COVID‐19 pandemic. It was found that anxiety and depression had a partial mediating effect.  These results suggest that staying in a good emotional state, developing an active coping style, and avoiding a passive coping style could decrease anxiety and depression, and further improve life satisfaction in medical workers.

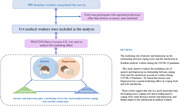

## 1 INTRODUCTION

The novel coronavirus disease 2019 (COVID‐19), which has persisted for over 2 years, has significantly impacted people's health and daily lives.^1^ Uncertain factors related to COVID‐19, such as duration, infection rate, and constantly changing policies, increased stress levels among individuals. Moreover, factors such as limited knowledge about the pandemic, the spread of rumors, fear of getting infected, and prolonged quarantine periods can harm mental health.

During the COVID‐19 pandemic, medical workers were required to cope with various situations with the possibility of infection. Medical workers were also concerned about the fear of infection in close relatives as they constantly came into contact with infected patients.^2^ Medical workers may suffer from acute stress, anxiety, depression, and insomnia.^3^, ^4^, ^5^ A meta‐analysis showed that the overall prevalence of depression and anxiety among healthcare workers during the COVID‐19 pandemic was 22.8% (ranging from 8.9% to 50.7%) and 23.2% (ranging from 10.4% to 44.7%), respectively.^6^ Moreover, the incidence of anxiety and depression among medical workers remained high even in the later stages of the pandemic.^7^ Anxiety and depression among medical workers affect their clinical work^8^ and have lasting adverse effects on their physical and mental health.^9^, ^10^, ^11^, ^12^, ^13^ All the studies mentioned above were survey studies using a specific cutoff and did not involve patients with confirmed diagnoses.

Coping style is defined as a way to manage stressful events while maintaining psychological balance.^14^ Coping styles include different coping strategies, generally categorized as active and passive.^15^, ^16^ Active coping strategies include accepting and learning from stressful events, overcoming stress, and making plans to solve problems. It is now understood that individuals with active coping styles are more prone to take constructive actions, including thinking positively and finding appropriate solutions. Individuals using active coping strategies deal with negative emotions by adopting positive cognition and seeking help, which benefits individuals in solving problems and ameliorating negative emotions such as anxiety and depression and is associated with higher well‐being.^17^ In contrast, passive coping strategies include refusing to acknowledge the existence of stressful events, running away from the present problem, and giving up trying to pursue goals, which strengthens the feeling of pressure. Individuals with a tendency to use passive coping strategies adopt passive ways when facing problems or difficulties, including smoking, drinking, and taking drugs that hinder the progress of problem resolution and prolong emotional distress.^18^ This coping style has been associated with the development of anxiety and depression^19^, ^20^ and may trigger mental disorders.^21^

Life satisfaction refers to “a person's overall evaluation of life quality according to the criteria he or she chooses.”^22^ It is an essential cognitive component of subjective well‐being, encompassing people's cognitive and emotional evaluation of life.^23^ Life satisfaction includes self‐satisfaction with living conditions, self‐satisfaction with life, a sense of gain in life,  hope to change life, and achieving ideals, all directly related to mental health.^24^, ^25^, ^26^ When people experience more pleasant and fewer unpleasant emotions, engage in exciting activities, and feel more happiness and less pain, they are satisfied with their lives and have a strong sense of happiness.^27^ It has been reported that improving life satisfaction during the pandemic could increase happiness.^28^ A higher level of life satisfaction is associated with more meaning in life and hope, which can assist people in coping with emergencies.^29^, ^30^

Previous studies have suggested that active coping styles facilitate life satisfaction.^31^ Even if stressful events trigger anxiety or depression, individuals with active coping styles are more likely to face and deal with problems, reducing anxiety and depression.^32^ In medical workers, active coping is reportedly effective in reducing anxiety and depression^20^, ^33^ and beneficial to mental health.^2^, ^20^, ^33^, ^34^, ^35^ On the other hand, a passive coping style has been found to increase anxiety and depression^20^, ^33^ and is associated with poor psychological health.^2^, ^34^ However, these studies did not examine the effect and extent of anxiety or depression on the relationship between coping styles and life satisfaction in medical workers. Therefore, the present study aimed to examine the role of anxiety or depression in the relationship between coping styles and life satisfaction. We hypothesized that anxiety and depression would mediate the relationship between coping styles and life satisfaction among frontline medical workers.

## 2 METHODS

### 2.1 Participants

Following the confirmation of COVID‐19 cases in Zunyi, medical workers diligently conducted COVID testing across different locations, such as communities, quarantine areas, cities, and rural areas. They worked under demanding conditions characterized by high intensity and constant exposure to the risk of infection. This study examined the associations among emotions, coping styles, and life satisfaction among medical staff in this context. A questionnaire‐based survey was conducted among these medical staff from October 28 to November 1, 2021. The inclusion criteria for the study were as follows: (1) medical workers aged between 18 and 60 years engaged in clinical work in the pandemic area; (2) voluntary participation in the survey; and (3) no history of neurological or psychiatric diseases. The exclusion criteria included: (1) a documented history of mental disorders; (2) non‐frontline medical staff; and (3) incomplete data collected from the online questionnaire. The medical ethics committee of Affiliated Hospital, Zunyi Medical University approved this study (Biomedical Research Ethics Committee, Affiliated Hospital of Zunyi Medical University, Approval number KLL‐2021‐311, approved on October 27, 2021) and all participants provided informed consent.

### 2.2 Procedure

The current study employed a cross‐sectional observational design, using an online questionnaire as the primary data collection method. The survey was conducted through Wenjuanxing, an online questionnaire survey platform (www.wjx.cn), from October 28 to November 1, 2021. Medical workers involved in clinical work on the frontline during this period were invited to participate. Instructions were provided to explain the purpose of the survey. The survey consisted of demographic information and the following questionnaires. An electronic informed consent form was provided, allowing individuals who wished to participate to consent and continue answering the questionnaires, while those who did not want to participate could quit directly.

### 2.3 Measures

The Self‐rating Anxiety Scale (SAS)^36^ includes 20 questions rated on a 4‐point scale, assessing the frequency of symptoms defined by the items. A scale score multiplied by 1.25 was used to obtain a standard score, where a standard score of less than 50 is considered normal, and 50 or above is considered clinically meaningful anxiety, used to calculate the prevalence of anxiety. The SAS has been proven to have good psychometric properties, with a Cronbach's alpha of 0.81 in this study.

The Self‐rating Depression Scale (SDS)^37^, ^38^ includes 20 questions rated on a 4‐point scale. The crude score was multiplied by 1.25 to obtain the standard score, where 53 is the cutoff for the SDS standard score, with 53 or higher indicating clinically meaningful depression, used to calculate the prevalence of depression.^39^, ^40^, ^41^, ^42^ The SDS has been proven effective and reliable, with a Cronbach's alpha of 0.75 in this study.

The Satisfaction with Life Scale (SWLS)^43^, ^44^ consists of 5 items scored on a 7‐point scale, where a higher score indicates better life satisfaction. The SWLS has been proven effective and reliable, with a Cronbach's alpha of 0.85 in this study.

The Simplified Coping Style Questionnaire (SCSQ)^18^ consists of 20 questions, including active and passive coping styles.^15^, ^16^ The active coping dimension (items 1–12) focuses on active coping characteristics, such as “Through work, study, or some other activity.” The passive coping dimension (items 13‐20) focuses on passive coping characteristics, such as “Relieving worries by smoking, drinking, taking drugs, or holding things.” The SCSQ has shown good reliability and validity,^18^ with a Cronbach's alpha of 0.88 for the active coping style subscale and 0.77 for the passive coping style subscale in this study.

### 2.4 Statistical analysis

The sample size for the study was calculated using MedCalc v20.0 statistical software (MedCalc Software bvba). Parameters such as statistical significance (*p* < 0.05), statistical power (0.8), expected area under the receiver operating characteristic (ROC) curve (0.9), and ROC value for inclusion of the null hypothesis (0.8) were set before plotting the ROC curve. Based on these parameters, a sample size of 505 participants was determined for the study. To ensure the accuracy of the results, a larger sample was targeted. As a result, 555 questionnaires were collected from anti‐epidemic medical personnel who voluntarily participated in the survey. From the pilot study, it was determined that questionnaires completed in more than 1 min could be considered valid. All participants were valid cases. Forty‐one participants who reported professions other than doctors or nurses were excluded, resulting in 514 valid questionnaires.

Data analysis was conducted in SPSS 24.0; Kolmogorov–Smirnov and Levene's tests were performed to examine the distribution and homogeneity of variances in anxiety, depression, life satisfaction, and coping styles. Given that the data exhibited normal distribution and homogeneity of variances, continuous variables were reported as mean standard deviation), while categorical variables were presented as the number of cases (*n*, %). Pearson correlation analysis was used to investigate the correlation among anxiety, depression, coping styles, and life satisfaction. Mediation analysis was performed using PROCESS Macro (version 4.0) in SPSS 24.0.^45^ Bootstrapping with 5,000 replications was used to estimate the standard errors (SEs) for effects. Coping styles (active coping or passive coping) were set as the independent variables, life satisfaction was set as the dependent variable, and anxiety or depression was set as the mediating variable. Only one independent variable and a mediating variable were included in each analysis. Effects were considered significant if the 95% confidence interval did not include 0. The significance level was set at 0.05 (two‐tailed).

## 3 RESULTS

### 3.1 Descriptive statistics of anxiety, depression, life satisfaction, and coping styles among medical workers

The study included 86 male and 428 female participants, comprising 178 doctors and 336 nurses. As shown in Table 1, 116 participants (22.57%) exhibited anxiety symptoms, while 94 participants (18.29%) showed signs of depression. Men displayed lower levels of anxiety and depression compared to women (*t* = −3.08, *p* = 0.002 and *t* = −3.21, *p* = 0.001, respectively). Similarly, doctors had lower levels of anxiety and depression than nurses (*t* = −4.06, *p* < 0.001, and t = −3.25, *p* = 0.001, respectively).

**Table 1:** Descriptive statistics of anxiety, depression, life satisfaction, and coping style among medical workers.

Variable	Males	Females	Doctors	Nurses	Total
Number	86 (16.73%)	428 (83.27%)	178 (34.6%)	336 (65.4%)	514 (100.00%)
Age	28.43 (5.38)	30.43 (5.86)	27.71 (5.42)	31.35 (5.64)	30.10 (5.82)
Education duration	18.28 (1.577)	17.24 (0.95)	18.25 (1.55)	16.97 (0.42)	17.41 (1.15)
SAS	40.06 (8.71)	43.30 (8.93)	40.59 (8.62)	43.91 (8.95)	42.76 (8.97)
SDS	38.34 (10.78)	42.61 (11.34)	39.68 (11.44)	43.07 (11.14)	41.90 (11.35)
SWLS	21.13 (9.60)	21.26 (8.76)	21.06 (9.42)	21.34 (8.62)	21.24 (8.90)
SCSQ_active	36.41 (6.91)	36.54 (6.74)	37.02 (6.54)	36.26 (6.88)	36.52 (6.76)
SCSQ_passive	17.13 (5.12)	17.77 (4.46)	17.45 (4.90)	17.78 (4.40)	17.66 (4.58)

Abbreviations: SAS, Self‐rating Anxiety Scale; SCSQ_active, the score of active coping style; SCSQ_passive, the score of passive coping style; SDS, Self‐rating Depression Scale; SWLS, Satisfaction with Life Scale.

### 3.2 Correlations among anxiety, depression, life satisfaction, and coping styles

Correlation analyses revealed that anxiety was positively correlated with depression (*r* = −0.695, *p* < 0.001). Furthermore, anxiety and depression were positively correlated with passive coping style (*r* = 0.258, *p* < 0.001 and *r* = 0.218, *p* < 0.001, respectively) and negatively correlated with life satisfaction (*r* = −0.261, *p* < 0.001 and *r* = −0.405, *p* < 0.001, respectively) and active coping styles (*r* = −0.278, *p* < 0.001 and *r* = −0.398, *p* < 0.001, respectively). Life satisfaction positively correlated with active coping styles (*r* = 0.294, *p* < 0.001). Interestingly, active coping style also displayed a positive correlation with passive coping style (*r* = 0.233, *p* < 0.001) (Table 2).

**Table 2:** Correlation analysis of anxiety, depression, life satisfaction, and coping styles.

	SAS	SDS	SWLS	SCSQ_active	SCSQ_passive
SAS	1				
SDS	0.695***	1			
SWLS	−0.261***	−0.405***	1		
SCSQ_active	−0.278***	−0.398***	0.294***	1	
SCSQ_passive	0.258***	0.218***	−0.073	0.233***	1

*Note*: ***p < 0.001.

Abbreviations: SAS, Self‐rating Anxiety Scale; SCSQ_active, the score of active coping style; SCSQ_passive, the score of passive coping style; SDS, Self‐rating Depression Scale; SWLS, Satisfaction with Life Scale.

### 3.3 The mediating role of anxiety or depression on the relationship between coping styles and life satisfaction

**Figure 1:**
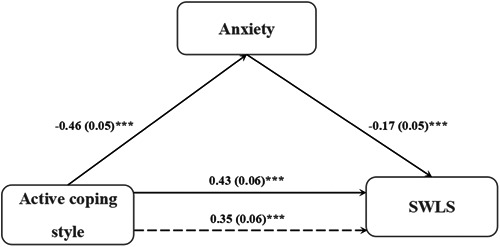
The mediating role of anxiety on the relationship between active coping style and life satisfaction. SWLS, Satisfaction With Life Scale. ***p < 0.001.

In this part of our analysis, the independent variable was active coping style, the dependent variable was life satisfaction, and anxiety served as the mediating variable. Covariates included gender, age, profession, years of education, and passive coping style. The results indicated that anxiety played a partial mediating role in the relationship between active coping style and life satisfaction (*R* ^2^ = 0.46, *F* = 22.87, *p* < 0.001). The indirect effect had a 95% confidence interval of (0.03–0.13) (Table 3 and Figure 1). Anxiety accounted for 18.60% of the effect of active coping style on life satisfaction.

**Table 3:** The mediating role of anxiety on the relationship between active coping style and life satisfaction.

Variables	Paths	Effect	SE	Bootstrap method 95% CI	
				Lower	Upper
Indirect effect	Active coping style→Anxiety→SWLS	0.08	0.03	0.03	0.13
Direct effect	Active coping style→SWLS	0.35	0.06	0.23	0.47
Total effect	Active coping style→SWLS	0.43	0.06	0.32	0.54

Abbreviations: CI, confidence interval; SE, standard error; SWLS, Satisfaction with Life Scale.

**Figure 2:**
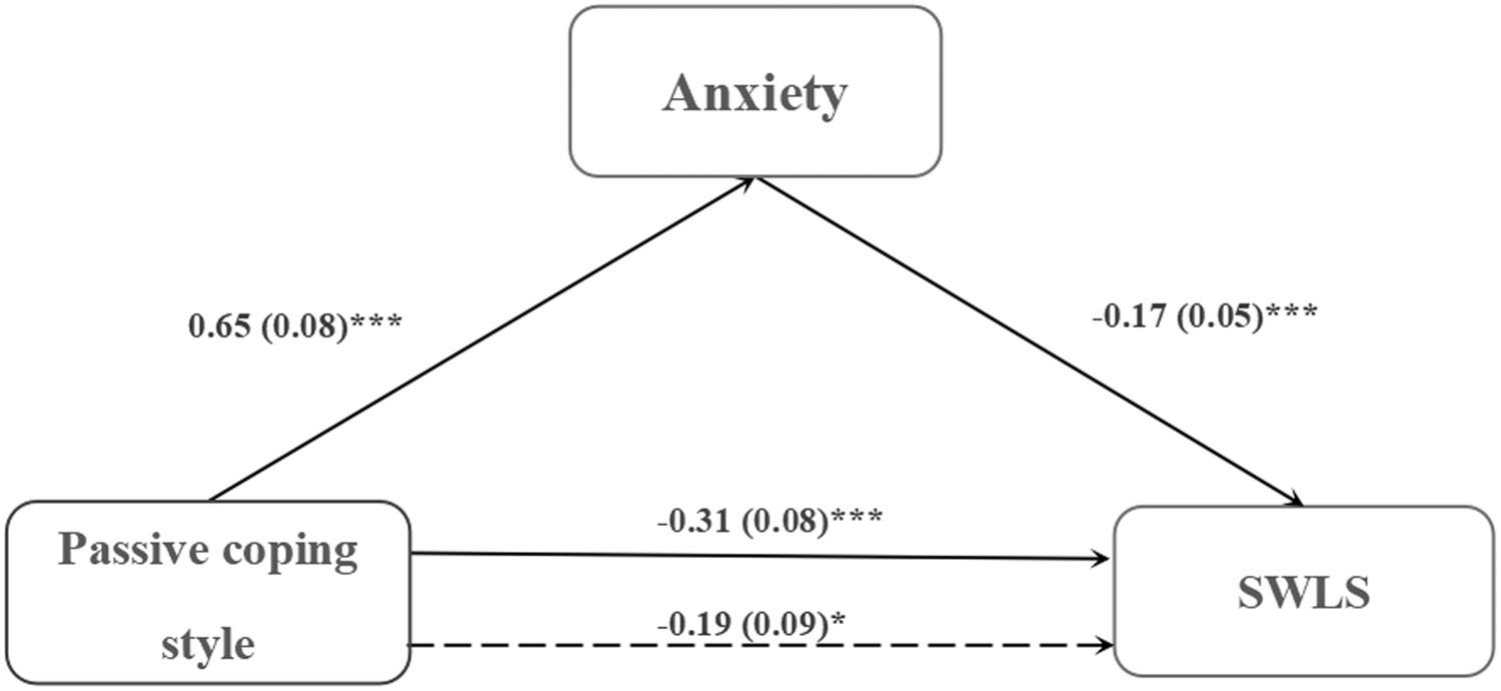
The mediating role of anxiety on the relationship between passive coping style and life satisfaction. SWLS, Satisfaction With Life Scale. *p < 0.05, ***p < 0.001.

Moreover, passive coping style was taken as the independent variable, life satisfaction as the dependent variable, and anxiety as the mediating variable; gender, age, profession, years of education, and active coping style were included as covariates. The results showed that anxiety played a partial mediating role in the relationship between passive coping style and life satisfaction (*R* ^2^ = 0.46, *F* = 22.87, *p* < 0.001). The indirect effect had a 95% confidence interval (−0.19 to −0.05) (Table 4 and Figure 2). Anxiety accounted for 35.48% of the effect of passive coping style on life satisfaction.

**Table 4:** The mediating role of anxiety on the relationship between passive coping style and life satisfaction.

Variables	Paths	Effect	SE	Bootstrap method 95% CI	
				Lower	Upper
Indirect effect	Passive coping style→Anxiety→SWLS	−0.11	0.04	−0.19	−0.05
Direct effect	Passive coping style→SWLS	−0.19	0.09	−0.37	−0.02
Total effect	Passive coping style→SWLS	−0.31	0.08	−0.47	−0.14

Abbreviations: CI, confidence interval; SE, standard error; SWLS, Satisfaction With Life Scale.

**Figure 3:**
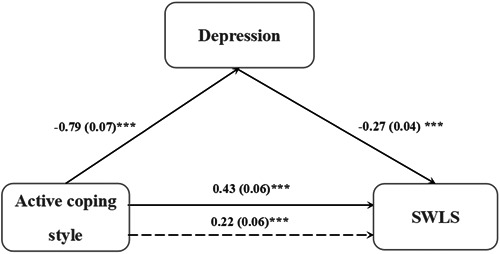
The mediating role of depression on the relationship between active coping style and life satisfaction. SWLS, Satisfaction With Life Scale. ***p < 0.001.

Next, active coping style was considered as the independent variable, life satisfaction as the dependent variable, and depression as the mediating variable; gender, age, profession, years of education, and passive coping style were included as covariates. The results revealed that depression played a partial mediating role in the relationship between active coping style and life satisfaction (*R* ^2^ = 0.53, *F* = 33.02, *p* < 0.001). The indirect effect had a 95% confidence interval (0.14–0.28) (Table 5 and Figure 3). Depression accounted for 48.84% of the effect of active coping style on life satisfaction.

**Table 5:** The mediating role of depression on the relationship between active coping style and life satisfaction.

Variables	Paths	Effect	SE	Bootstrap method 95% CI	
				Lower	Upper
Indirect effect	Active coping style→Depression→SWLS	0.21	0.04	0.14	0.28
Direct effect	Active coping style→SWLS	0.22	0.06	0.10	0.34
Total effect	Active coping style→SWLS	0.43	0.06	0.32	0.54

Abbreviations: CI, confidence interval; SE, standard error; SWLS, Satisfaction with Life Scale.

**Figure 4:**
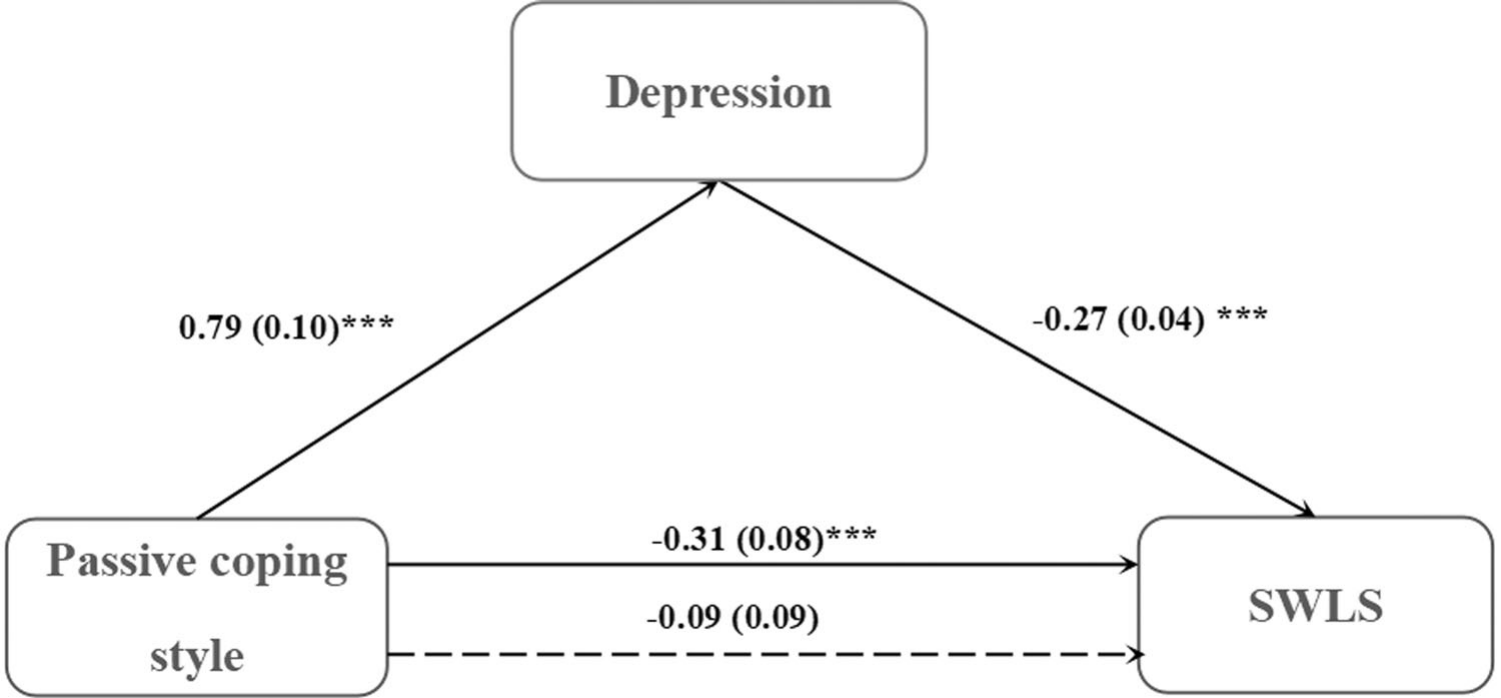
The mediating role of depression on the relationship between passive coping style and life satisfaction. SWLS, Satisfaction With Life Scale. ***p < 0.001.

Finally, passive coping style was taken as the independent variable, life satisfaction as the dependent variable, and depression as the mediating variable; gender, age, profession, years of education, and active coping style were included as covariates. The results demonstrated that depression mediated the relationship between passive coping style and life satisfaction (*R* ^2^ = 0.53, *F* = 33.02, *p* < 0.001). The indirect effect had a 95% confidence interval (−0.31 to −0.14) (Table 6 and Figure 4). Depression accounted for 67.74% of the effect of passive coping style on life satisfaction.

**Table 6:** The mediating role of depression on the relationship between passive coping style and life satisfaction.

Variables	Paths	Effect	SE	Bootstrap method 95% CI	
				Lower	Upper
Indirect effect	Passive coping style→Anxiety→SWLS	−0.21	0.04	−0.31	−0.14
Direct effect	Passive coping style→SWLS	−0.09	0.09	−0.26	−0.07
Total effect	Passive coping style→SWLS	−0.31	0.08	−0.47	−0.14

Abbreviations: CI, confidence interval; SE, standard error; SWLS, Satisfaction with Life Scale.

## 4 DISCUSSION

In the present study, we found that the prevalence of anxiety among frontline medical workers during the COVID‐19 pandemic in Zunyi was 22.57%, while the prevalence of depression was 18.29%. Females exhibited higher levels of anxiety and depression compared to males, and nurses showed higher levels of anxiety and depression compared to doctors. Anxiety and depression were positively correlated with passive coping style but negatively correlated with life satisfaction and active coping style. On the other hand, life satisfaction was positively correlated with active coping style but negatively correlated with passive coping style. Mediation analyses revealed that anxiety and depression mediated the relationship between coping styles and life satisfaction. In this respect, anxiety accounted for 18.6% of the variance in the effect of active coping style on life satisfaction, while passive coping style accounted for 35.48%. Depression accounted for 48.84% of the variance in the effect of active coping style and 67.74% of the effect of passive coping style on life satisfaction. To our knowledge, this study is the first to examine the role of anxiety and depression in the relationship between coping styles and life satisfaction among frontline medical workers in the pandemic area during the COVID‐19 crisis.

### 4.1 Anxiety, depression, life satisfaction, and coping styles of frontline medical workers

As mentioned above, the prevalence of anxiety among frontline medical workers during the pandemic in Zunyi was 22.57%, while the prevalence of depression was 18.29%, consistent with the literature.^3^, ^4^, ^5^, ^6^, ^7^, ^8^, ^9^, ^10^, ^11^, ^12^, ^46^, ^47^ These negative emotions may be attributed to acute stress, such as the urgency of completing nucleic acid tests and concerns about personal safety.^48^ The prevalence of anxiety and depression in Zunyi was lower than in other countries. For instance, in Oujda, 71.1% of medical workers were afraid of contracting COVID‐19, 97.6% were afraid of spreading the virus to relatives, more than two‐thirds of the respondents described negative feelings and exhaustion, 49.4% had a low sense of well‐being and experienced depressive symptoms, and 67% reported high levels of stress.^47^ Similarly, in Finland,^46^ 45% of hospital workers experienced anxiety, while in Saudi Arabia,^49^ nearly 30% of medical workers suffered from depression (45% of hospital workers had anxiety). Moreover, the prevalence of anxiety and depression in Zunyi was lower than in certain areas in China, such as Jinan (anxiety prevalence of 28.5% and depression prevalence of 56.0%)^50^ and Xinjiang (49.6% and 60.2% for anxiety and depression, respectively).^3^ However, it was higher than in some countries, such as Turkey, where the overall prevalence of depression was 15.4%. It was also higher than in certain areas in China, where the proportions of anxiety and depression among medical workers in tertiary hospitals in central and southern China were 10.81% and 6.13%, and 18.56% and 11.34% among medical workers, respectively.^51^ Although the prevalence of anxiety in the present study was similar to a meta‐analysis (22.57 vs. 23.2%), the prevalence of depression was slightly lower (18.29 vs. 22.8%).^6^ The different prevalence rates may be attributed to various factors, such as the timing of the studies during the pandemic,^52^ regional and national differences,^53^ cultural factors,^54^ occupations,^55^ and pandemic fatigue.^56^

In agreement with previous studies, females scored higher on anxiety and depression than males, which suggested that women tend to experience higher levels of anxiety and depression and are more likely to be affected by these conditions.^57^, ^58^, ^59^, ^60^ Additionally, our study revealed that nurses had a higher level of anxiety and depression than doctors. This finding may be associated with the higher psychological resilience observed among doctors than nurses.^33^ Resilience has been also shown to be beneficial in mitigating the adverse effects of the pandemic on mental health.^61^, ^62^

### 4.2 Correlations among anxiety, depression, life satisfaction, and coping styles

The study findings revealed a positive correlation between anxiety and depression, consistent with previous research, indicating a significant overlap between these two emotional states and emotional disorders.^63^ A significant negative correlation has been reported between psychological stress and active coping style, and a significant positive correlation was documented between psychological stress and passive coping style among obstetric nurses.^64^ Similarly, anxiety and depression have been found to have a significant negative correlation with active coping style and a significant positive correlation with passive coping style.^65^, ^66^ Active coping style has been suggested to provide emotional protection for nurse practitioners,^67^ while lower scores in avoidant coping, higher levels of optimism, and greater resilience have been associated with reduced stress, anxiety, and depressive symptoms in medical residents.^19^ Additionally, studies have shown that higher levels of life satisfaction are associated with a greater sense of meaning and hope in life, which can aid individuals in coping with emergencies.^29^, ^30^ Passive coping style (specifically, avoidant coping) significantly mediated the relationship between stress related to the pandemic and various psychosocial outcomes, with depression as the epicenter. However, they did not examine the relationship between life satisfaction and coping styles among medical workers, providing additional insights by demonstrating a positive correlation between life satisfaction and active coping style, and a negative correlation between life satisfaction and both anxiety/depression and passive coping style. Interestingly, we found a positive correlation between active coping style and passive coping style. Although slightly counterintuitive, we propose that individuals who adopt more active coping strategies may also engage in passive coping to some extent, despite active coping being associated with positive outcomes and passive coping being related to negative outcomes.

### 4.3 The mediating role of anxiety and depression in the relationship between coping styles and life satisfaction

Previous studies have established an association between anxiety, depression, psychological well‐being, and life satisfaction.^68^ Moreover, coping style significantly affects anxiety, depression,^32^ and life satisfaction.^69^ Problem‐focused strategies, which represent an active coping style, have been linked to improved well‐being.^17^ However, these studies did not investigate the role of anxiety and depression in mediating the relationship between coping styles and life satisfaction. Our research utilized mediation analyses and revealed that anxiety and depression mediate the relationship between coping styles and life satisfaction. Specifically, anxiety and depression negatively mediate the connection between active coping styles and life satisfaction since active coping styles have a protective effect in helping medical workers adjust to negative emotions,^67^ and promote the mental well‐being of medical professionals.^20^, ^33^, ^34^, ^70^ In contrast, anxiety and depression positively mediated the relationship between passive coping styles and life satisfaction. This suggests that passive coping styles may intensify negative emotions among medical workers, subsequently reducing their overall life satisfaction.^20^, ^33^, ^34^, ^70^ These results are consistent with a study demonstrating that avoidant coping (a form of passive coping style) mediates the relationship between pandemic‐related stress and psychosocial outcomes, especially depression.^71^

### 4.4 Limitations and implications

Several limitations of the current study should be acknowledged. First, the study employed an online survey method, which did not incorporate lie‐detection items. However, individuals with short response times were excluded from the analysis. Second, the study design was cross‐sectional, thus limiting the ability to establish causal relationships. Future longitudinal studies can examine the stability of the associations between these variables over time. Third, the study exclusively focused on medical workers whose mental health concerns may not be directly comparable to the general public, warranting further investigation. Lastly, the positive correlation between active and passive coping styles requires further examination, as both styles are not inherently good or bad. Future studies should explore their specific effects on mental health.

Despite these limitations, the study provided insights into the emotional state, life satisfaction, coping styles, and interrelationships among frontline medical workers who transitioned from low‐risk areas to pandemic areas during the outbreak. Significantly, this study identified the mediating effects of depression and anxiety on the relationship between coping styles and life satisfaction among frontline medical workers for the first time. Future research should investigate mental well‐being dynamics in greater detail to improve mental health interventions.

## 5 CONCLUSIONS

The findings of this study indicate that a proportion of medical workers experienced anxiety and depression during the pandemic. Anxiety and depression were positively correlated with passive coping style but negatively correlated with life satisfaction and active coping style. Notably, life satisfaction exhibited a significant positive association with active coping style. Furthermore, anxiety and depression mediated the relationship between coping styles and life satisfaction. Anxiety accounted for 18.6% of the effect of active coping style and 35.48% of the effect of passive coping style on life satisfaction. Likewise, depression accounted for 48.84% of the effect of active coping style and 67.74% of the effect of passive coping style on life satisfaction. These findings suggest that fostering positive coping styles while avoiding passive coping styles may contribute to reducing anxiety and depression and enhancing life satisfaction among medical workers.

## AUTHOR CONTRIBUTIONS

Gui‐Fang Chen, Chang‐Yin Yu, and Jun Zhang completed the concept and research design; Gui‐Fang Chen and Juan Peng collected the data; Gui‐Fang Chen, Ye‐Ping Zhang, Xin‐Lan Zhang, and Jun Liu analyzed the data; Gui‐Fang Chen, Ye‐Ping Zhang, and Zhi‐Jie Wei wrote the first draft; Chang‐Yin Yu, Jun Zhang, and Zu‐Cai Xu revised the manuscript.

## CONFLICT OF INTEREST STATEMENT

Zu‐Cai Xu is Associate Editor of Ibrain Journal editorial board. He is not involved in the peer review and editorial decision‐making processes of this article. The remaining authors declared they have no competing interests. [Correction added on 9 December 2023 after first online publication: This section was revised at the request of authors.]

## ETHICS STATEMENT

This study was approved by the Biomedical Research Ethics Committee of Affiliated Hospital of Zunyi Medical University (Approval number KLL‐2021‐311). Participants had signed an electronic informed consent form. [Correction added on 9 December 2023 after first online publication: This section was revised at the request of authors.]

## Data Availability

The data supporting the conclusions of this study will be made available by the corresponding author on reasonable request.
